# Viral Evasion of RIG-I-Like Receptor-Mediated Immunity through Dysregulation of Ubiquitination and ISGylation

**DOI:** 10.3390/v13020182

**Published:** 2021-01-26

**Authors:** Cindy Chiang, Guanqun Liu, Michaela U. Gack

**Affiliations:** Florida Research and Innovation Center, Cleveland Clinic, Port Saint Lucie, FL 34987, USA; CHIANGC2@ccf.org (C.C.); LIUG2@ccf.org (G.L.)

**Keywords:** viral evasion, ubiquitin, ISG15, innate immunity, interferon

## Abstract

Viral dysregulation or suppression of innate immune responses is a key determinant of virus-induced pathogenesis. Important sensors for the detection of virus infection are the RIG-I-like receptors (RLRs), which, in turn, are antagonized by many RNA viruses and DNA viruses. Among the different escape strategies are viral mechanisms to dysregulate the post-translational modifications (PTMs) that play pivotal roles in RLR regulation. In this review, we present the current knowledge of immune evasion by viral pathogens that manipulate ubiquitin- or ISG15-dependent mechanisms of RLR activation. Key viral strategies to evade RLR signaling include direct targeting of ubiquitin E3 ligases, active deubiquitination using viral deubiquitinating enzymes (DUBs), and the upregulation of cellular DUBs that regulate RLR signaling. Additionally, we summarize emerging new evidence that shows that enzymes of certain coronaviruses such as SARS-CoV-2, the causative agent of the current COVID-19 pandemic, actively deISGylate key molecules in the RLR pathway to escape type I interferon (IFN)-mediated antiviral responses. Finally, we discuss the possibility of targeting virally-encoded proteins that manipulate ubiquitin- or ISG15-mediated innate immune responses for the development of new antivirals and vaccines.

## 1. Introduction

### 1.1. RIG-I-Like Receptors and Their Signaling Pathway

Infectious diseases caused by viruses affect the global population at an alarming rate, as can be seen by the current COVID-19 pandemic that has caused millions of infections worldwide. Given the large number of emerging and re-emerging viruses that exist, it is important to understand how these pathogens successfully replicate in the human host and how they induce disease. A hallmark of successful viral pathogens is their potent ability to antagonize or dysregulate human innate immune responses, in particular the induction of antiviral and proinflammatory cytokines. The first step in innate immunity is the detection of the virus where innate immune sensors, also known as pattern-recognition receptors (PRRs), recognize virus-derived molecular signatures (called pathogen-associated molecular patterns, or PAMPs) that are distinct from the host, such as viral nucleic acids (viral RNA or DNA). In addition, mislocalized, unmasked, or misprocessed cellular RNA (mostly noncoding RNAs) and DNA (e.g., mitochondrial DNA) can also trigger certain types of PRRs, leading to activation of innate and intrinsic defense mechanisms [1].

A number of PRRs have been discovered, each recognizing unique and defined PAMPs. This review focuses on the family of retinoic acid-inducible gene-I (RIG-I)-like receptors (RLRs), which sense cytoplasmic RNA. The two major members of the RLR family are RIG-I and melanoma differentiation-associated protein 5 (MDA5), which trigger an antiviral program following the detection of distinct RNA agonists (Reviewed in [2,3,4]). Canonically, RIG-I detects quite short, hairpin, double-stranded RNA (dsRNA) that possesses a triphosphate or diphosphate moiety at the 5′ terminus. RIG-I also detects several cellular RNA polymerase III transcripts, such as the pseudogene transcripts of the cellular *5S* ribosomal RNA (i.e., *RNA5SP141*) when these are mislocalized in the cytoplasm and ‘unmasked’ due to downregulation of their binding proteins [5]. MDA5 was shown to detect longer dsRNA or aggregated RNA; however, physiological RNA agonists of MDA5 during virus infection still remain enigmatic. Virus infection experiments in *RIG-I* and/or *MDA5* gene-edited cells revealed that RIG-I senses a variety of negative-strand RNA viruses (e.g., influenza (IAV) and vesicular stomatitis viruses (VSV)) as well as some herpesviruses (for example, herpes simplex virus-1 (HSV-1), Epstein–Barr virus (EBV), and Kaposi’s sarcoma herpesvirus (KSHV)), while MDA5 detects infection by picornaviruses and coronaviruses [6,7]. An increasing number of studies have, however, indicated that many RNA viruses are sensed by both RIG-I and MDA5 (Reviewed in [4]).

Both RIG-I and MDA5 are DExD/H-box-containing helicases possessing two N-terminal caspase activation and recruitment domains (CARDs), a large helicase domain, and a carboxyl-terminal domain (CTD). These structural components are necessary for RNA binding and interacting with the common adaptor mitochondrial antiviral signaling protein (MAVS). MAVS acts as a scaffold for RLR signaling by coordinating the recruitment and activation of TANK-binding kinase 1 (TBK1) and inhibitor of nuclear factor κB kinase (IKK), which then phosphorylate IRF3 and IRF7 (among other innate signaling proteins) [8]. The IRF proteins translocate from the cytosol to the nucleus to induce, together with other transcription factors, the gene expression of type I or type III interferons (IFNs), proinflammatory cytokines, and chemokines (Reviewed in [2]). Cytokines and chemokines are then secreted from the infected host cell, alarming other cells of the viral attack. In the case of type I or type III IFNs, engagement with their respective receptors on the surface of neighboring cells induces a signaling cascade that leads to the upregulation of many antiviral molecules, among them the gene products encoded by IFN-stimulated genes (ISGs). Effector proteins encoded by ISGs have many different mechanisms to block virus entry or replication (for example, some degrade viral RNA or inhibit the formation of viral replication complexes). Together, IFN-induced effector proteins create a cellular milieu that is hostile to the virus, thereby dampening viral replication in the host organism and virus spread.

### 1.2. Regulation of RLR Activity by Ubiquitin and ISG15 Modifications

Innate immune signaling must be tightly controlled to prevent excessive inflammatory responses and potential tissue damage. This is achieved through post-translational modifications (PTMs) that regulate each step of the antiviral innate immune response in a dynamic and complex way. They regulate the activity, subcellular localization, and/or protein-protein interactions of sensor and signaling proteins as well as key transcription factors in innate immunity. PTMs that are important for regulating IFN-mediated antiviral responses include the enzymatic addition of phosphate groups (phosphorylation), ubiquitin (ubiquitination), ISG15 (ISGylation), and acetylation, along with other less well-studied modifications like glutamylation and deamidation (Reviewed in [1,9]). Notably, all these PTMs are reversible, which allows for the dynamic regulation of protein function.

During ubiquitination, ubiquitin moieties, either as a single unit or in multimeric chains, are covalently attached to a target substrate via three enzymes: ubiquitin activating (E1), ubiquitin conjugating (E2), and ubiquitin ligase (E3) enzymes. E3 ligases are primarily responsible for substrate specificity of this PTM. Seven polyubiquitin-linkage types have been discovered (K6-, K11-, K27-, K33-, K48-, and K63-polyubiquitin linkages), each with its own distinct function to regulate the activity or abundance of target proteins. K48-linked ubiquitination and K63-linked ubiquitination are the linkage types most relevant to RLR regulation, and their roles in innate immunity have been extensively studied over the past several years. Modification with K48-linked polyubiquitin chains ‘earmarks’ the substrate protein for proteasomal degradation, while K63-linked ubiquitination (which is usually non-degradative) facilities the transduction of immune signaling. In many cases, K63-linked ubiquitin chains allow for the multimerization of sensor or signaling proteins, and the formation of larger signaling platforms. Ubiquitination is removed from the substrate by deubiquitinating enzymes (DUBs), which allows for an “on/off” switch to regulate innate immune signaling (Reviewed in [1,10]). Moreover, noncovalent (also called ‘unanchored’) ubiquitination plays an emerging role in the regulation of innate immunity [11,12].

ISGylation is a PTM in which the ubiquitin-like protein ISG15 (IFN-stimulated gene 15) is conjugated to a target protein also via E1, E2, and E3 enzymes (Reviewed in [13,14,15]); of note, only a few E3 ligases that catalyze ISGylation have been identified to date. ISG moieties can reversibly be removed by the deISGylating enzyme UBP43 (USP18), which also acts as an important negative regulator of type I IFN receptor (IFNAR) signaling during viral infection [16,17]. While *Usp18^-/-^* mice and cells from these mice displayed restricted virus replication after VSV or Sindbis virus (SINV) challenge [16,17], the generation of a USP18 knock-in mouse lacking isopeptidase activity (C61A) helped dissect the dual functions of USP18 and demonstrated host resistance to VSV, vaccinia virus, and influenza B virus specifically via enhanced ISG15 conjugation with unaltered IFNAR signaling [18]. Studies further clarified the role of USP18 in desensitizing IFNAR signaling by demonstrating the stabilization of USP18 by unconjugated ISG15, which, like ubiquitin, can bind to substrates noncovalently [13,19,20,21]. Nonetheless, covalent ISG15 modification, or ISGylation, is well known to play an antiviral role, restricting viruses from diverse families. Whereas ISGylation of specific viral proteins provides a mechanism for the antiviral role of ISG15 for specific viral infections, general host mechanisms that could explain the wide-spectrum antiviral activity of ISG15 have remained unknown until recently with the discovery of ISGylation of the sensor MDA5 [22].

A growing number of studies have demonstrated RLR regulation by a diverse set of PTMs. Dynamic acetylation and deamidation regulate dsRNA binding of RLRs [23,24,25]. The role of K63-linked ubiquitination is most well characterized for RLR function where K63-polyubiquitin chains located in the CARDs and CTD regulate RLR oligomerization-dependent signaling and auto-repression. Similarly, MDA5 ISGylation in the CARDs and K63-linked ubiquitination in the helicase domain are required for oligomerization and signaling [26,27].

### 1.3. Viral Evasion of Innate Immune Responses

**Figure 1:**
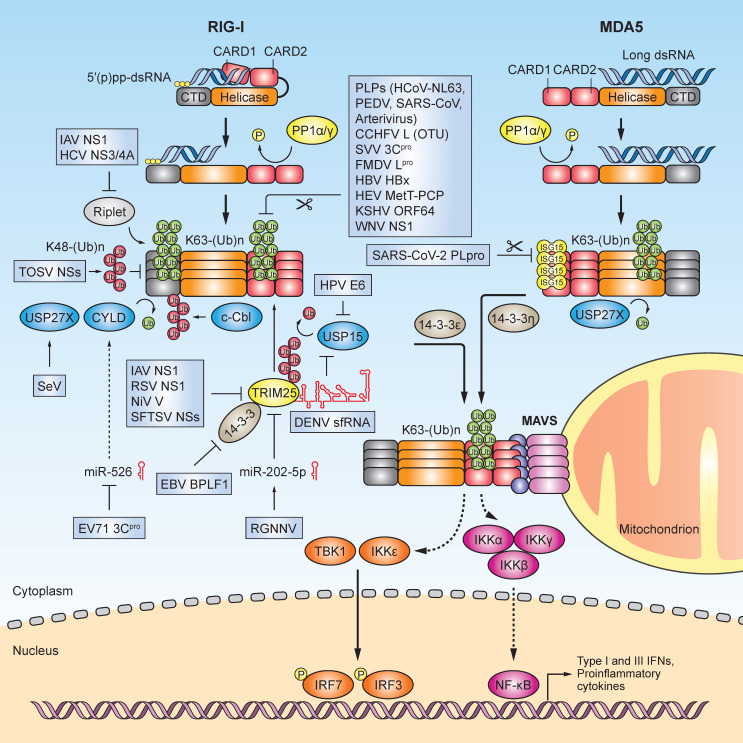
Evasion of RLR-mediated innate immunity through viral manipulation of ubiquitination and ISGylation. Schematic of the signaling pathway triggered by the innate immune sensors RIG-I and MDA5 upon recognition of host or viral immunostimulatory RNA. Activation of this pathway culminates with the interaction of RLRs with the shared adaptor protein mitochondrial antiviral signaling protein (MAVS) at the mitochondrion via specific 14-3-3 chaperone proteins (14-3-3ε and 14-3-3η). MAVS recruits the inhibitor of nuclear factor κB kinase (IKK)-related serine/threonine kinases TBK1 and IKKε, which phosphorylate and activate IRF3/7 (among other signaling mediators); these transcription factors then translocate from the cytoplasm to the nucleus to induce the gene expression of type I and type III IFNs, proinflammatory cytokines, and chemokines. RLRs also induce the NF-κB signaling program via IKKα/β/γ activation. The RLR-mediated antiviral response is tightly controlled by several reversible post-translational modifications (PTMs) including ubiquitination and ISGylation, which serve as “on/off” switches for signaling. E3 ligases such as TRIM25 and Riplet modify RIG-I with covalent K63-linked ubiquitin chains, which promotes RIG-I oligomerization and activation. However, viruses have evolved numerous mechanisms to antagonize RIG-I ubiquitination, which ultimately suppresses cytokine induction and blunts the antiviral response. The K63-linked ubiquitination of RIG-I is directly inhibited by numerous viral proteins such as CCHFV L (OTU), FMDV L^pro^, KSHV ORF64, WNV NS1, SVV 3C^pro^, HBV HBx, HEV MetT-PCP, and the papain-like proteases (PLPs) from HCoV-NL63, PEDV, and SARS-CoV. Viral proteins such as IAV NS1, RSV NS1, NiV V, HCV NS3/4A, and SFTSV NSs target TRIM25 and/or Riplet to blunt the K63-linked ubiquitination of RIG-I. EBV BPLF acts as a viral deconjugase to inhibit 14-3-3 and to sequester TRIM25 into aggregates. EV71 3C^pro^ downregulates miR-526, which targets the DUB enzyme CYLD, while RGNNV infection upregulates miR-202-5p, which blocks the TRIM25 function. The sfRNA from an epidemic strain of DENV interacts with TRIM25 and impedes its deubiquitination and stabilization by USP15, a cellular DUB. The E6 oncoproteins of several HPVs (both low-risk and high-risk) form a complex with TRIM25 and USP15, which ultimately prevents the K63-linked ubiquitination of the RIG-I CARDs. TOSV NSs induces K48-linked ubiquitination of RIG-I, promoting its destruction by the proteasome. USP27X is a negative regulator of IFN-β induction, and upon SeV infection, it removes K63-linked ubiquitination from both RIG-I and MDA5. MDA5 is modified by ISGylation at the CARDs (at K23 and K43), which promotes MDA5 oligomerization and activation; however, SARS-CoV-2 PLpro physically interacts with MDA5 and inhibits its CARD ISGylation, suppressing MDA5-mediated antiviral immunity.

The innate immune response mediated by RLRs elicits defense against a multitude of viruses including coronaviruses, influenza viruses, flaviviruses, rhabdoviruses, paramyxoviruses, and picornaviruses [28]. However, viruses have adopted evasion mechanisms to target RLRs in order to counteract or evade innate immune defenses. These strategies promote effective infection of the host in a variety of ways, whether it be by cleavage or degradation of PRRs or their signaling mediators, sequestration or modification of viral RNA-ligands, or manipulation of PTMs that regulate RLR activity (Reviewed in [29]). Studies to dissect each virus’ mode(s) of evasion are important for the development of antivirals and vaccines against newly emerging viruses or viruses for which no treatment exists. Viruses are capable of evading not only RLRs, but many other immune surveillance systems such as Toll-like receptors and the cGAS-STING pathway, which has been reviewed elsewhere [29,30,31,32]. In this review, we specifically summarize how viruses manipulate ubiquitination and ISGylation events to evade RLR-mediated innate immune signaling (Figure 1).

## 2. Viral Evasion of Ubiquitin-Mediated RLR Responses

Reversible ubiquitination regulates important steps of the RLR-signaling cascade (Reviewed in [1]). As such, viruses have usurped host control of the ubiquitination-dependent signaling events to blunt the IFN response and productively infect the cell by a variety of mechanisms. There are many examples of viral manipulation of ubiquitination within the context of RLR-mediated immunity, acting on multiple signaling molecules crucial to the pathway. Below we will discuss: (i) Viral targeting of ubiquitin E3 ligases; (ii) Inhibition of RLRs by virus-encoded DUBs; (iii) Induction of host DUBs critical for RLR regulation; and (iv) Virus-induced degradation of RLRs through K48-linked polyubiquitination.

### 2.1. Viral Targeting of Ubiquitin E3 Ligases

Common targets of viral antagonism are the cellular E3 ligase enzymes that activate RIG-I by catalyzing K63-linked ubiquitination. One critical positive regulator of RIG-I-mediated innate immune signaling is the E3 ligase TRIM25, which attaches K63-linked ubiquitin chains to the RIG-I CARDs [33,34]. Upon RNA recognition by RIG-I, TRIM25 interacts with the first CARD of RIG-I and attaches a K63-linked ubiquitin chain to the K172 residue in the second CARD. Structural analysis highlighted that TRIM25 RING domain dimerization leads to higher-order oligomerization and catalytic activation of TRIM25, which is required for RIG-I ubiquitination and subsequent activation [35]. Recent studies have shown that TRIM25 also possesses RNA-binding activity, via its PRY/SPRY domain and coiled-coil domain (CCD), which regulates the ubiquitin E3 ligase activity of TRIM25, its oligomeric state, localization within the cell, and antiviral activity [36,37,38]. Several viruses have evolved to antagonize the TRIM25-RIG-I signaling axis, thereby limiting IFN responses (Figure 1).

IAV nonstructural protein 1 (NS1) inhibits the RIG-I-mediated IFN response through a direct interaction with TRIM25. NS1 was first shown to interact with the CCD of TRIM25, preventing TRIM25 dimerization and, thereby, its enzymatic activity. This study also revealed that the E96/E97 residues in NS1, which are located in a highly conserved protein-protein-interacting motif, were required for TRIM25 binding and efficient suppression of RIG-I CARD ubiquitination catalyzed by TRIM25 [39]. More recently, it was shown via crystal structures of interacting TRIM25 CCD and IAV NS1 that NS1 binding interferes with the positioning of the C-terminal TRIM25 PRY/SPRY domain, which prevents the K63-linked ubiquitination of RIG-I [40]. Further studies revealed that the NS1 proteins from human and avian IAV strains interact with TRIM25 in a species-specific manner, and that the NS1 of some IAV strains also binds Riplet [41], which is another major E3 ligase that modifies RIG-I with K63-linked ubiquitin chains [42].

NS1 proteins of other negative-strand RNA viruses also target TRIM25. Respiratory syncytial virus (RSV) NS1 protein directly binds to TRIM25 at its PRY/SPRY domain. This interaction suppresses the K63-linked ubiquitination of the RIG-I CARDs and disrupts their interaction with the MAVS CARD. Ectopic expression of TRIM25 reverses evasion by RSV NS1 and recoups IFN induction [43]. The V proteins of certain paramyxoviruses such as Nipah virus (NiV) and parainfluenza virus, which are well known to inhibit MDA5 signaling [44,45,46], have recently been shown to interact with both RIG-I (via its CARDs) and TRIM25 (via its C-terminal PRY/SPRY domain). Complex formation of V protein with TRIM25 and RIG-I prevents the K63-linked ubiquitination of the RIG-I CARDs and downstream IFN-β gene expression [47]. West Nile virus (WNV) NS1 decreases the K63-linked ubiquitination of the sensor RIG-I, although it directly binds to RIG-I rather than to TRIM25. WNV NS1 binding to the related sensor MDA5 and a reduction of MDA5 protein expression was also observed; however, no further study was performed to determine whether degradative ubiquitination of MDA5 was responsible for this phenomenon [15].

Recently, mass spectrometry analysis showed that the nonstructural protein NSs of severe fever with thrombocytopenia syndrome (SFTSV), a member of the large family *Bunyaviridae*, interacts with TRIM25 but not Riplet or RIG-I. NSs inhibits the K63-linked ubiquitination of RIG-I by relocalizing and trapping TRIM25 in viral inclusion bodies. Furthermore, a PXXP motif at amino acid residues 66−69 is known to be important for NSs function, and mutations at these residues abrogated the ability of NSs to block the TRIM25-mediated K63-linked ubiquitination of RIG-I [48].

Abrogation of Riplet-mediated K63-linked ubiquitination of the CTD of RIG-I has been reported for hepatitis C virus (HCV) nonstructural protein 3/4A (NS3/4A) [49], which is a serine protease that blocks IFN-β production via direct cleavage of MAVS [50,51]. The K63-linked ubiquitination of RIG-I by Riplet was abrogated by HCV NS3/4A when overexpressed. Interestingly, this reduction was accompanied with a loss of interaction between TRIM25 and RIG-I. Studies with an HCV replicon showed that the virus targets endogenous Riplet and blocks the K63-linked ubiquitination of RIG-I in Huh7 cells [49].

Not only viral proteins but also viral RNAs bind to TRIM25 to antagonize RIG-I signaling. Subgenomic flavivirus RNA (sfRNA) is a noncoding RNA derived from the 3′ untranslated region of the flavivirus genome and the product of incomplete degradation by the cellular ribonuclease XRN1 [52]. It is also unique to the family *Flaviviridae* and required for viral pathogenicity [53,54]. The sfRNA from a dengue virus (DENV) serotype 2 strain that arose from an epidemic in Puerto Rico in 1994 (strain PR-2B) interacts with human TRIM25 and inhibits its deubiquitination and likely protein stability. The protein abundance of TRIM25 is dynamically regulated by degradative K48-linked ubiquitination and deubiquitination, the latter being catalyzed by the ubiquitin-specific peptidase 15 (USP15). Thus, USP15 stabilizes TRIM25 and allows it to effectively ubiquitinate and activate RIG-I [55]. However, in cells infected with the strain PR-2B of DENV-2, sfRNA immunoprecipitated with TRIM25 and this interaction decreased TRIM25 deubiquitination and downstream IFN induction by RIG-I [56].

Another virus that targets the USP15-TRIM25-RIG-I axis is human papilloma virus (HPV), which encodes the oncoprotein E6. HPV E6 binds to both TRIM25 and USP15 when ectopically expressed or in natively HPV-infected cells. Co-Immunoprecipitation studies indicated that the interaction between TRIM25 and E6 was conserved in high-risk (HPV18, HPV33, and HPV52), low-risk (HPV6 and HPV11), and cutaneous beta genus type (HPV5 and HPV8) HPVs. This interaction promotes TRIM25 ubiquitination and degradation, suppresses K63-linked ubiquitination of RIG-I, and blocks the RIG-I-MAVS interaction, ultimately dampening RIG-I signaling [57] (Figure 1). These data also provide evidence that RIG-I is important for the sensing of small DNA viruses such as HPV.

### 2.2. Virus-Encoded DUB Enzymes

Many studies have demonstrated that several virus-encoded proteins have the ability to act as DUBs to remove ubiquitin moieties from RIG-I. Arteriviruses that, along with Coronaviruses and Roniviruses, comprise the *Nidovirales* order, have DUB activity via their nonstructural protein 2 (nsp2), which belongs to the papain-like protease (PLP) family. Nidovirus PLPs are multifunctional in that they serve as proteases, DUBs, and deISGylases [58]. Viral PLP DUB activity is retained by all members of the Arterivirus family including equine arteritis virus (EAV), porcine respiratory and reproductive syndrome virus (PRRSV), lactate dehydrogenase-elevating virus (LDV), and simian hemorrhagic fever virus (SHFV), although LDV PLP appears to be less efficient. The enzymatic activity of nsp2 was critical to remove K48- and K63-linked ubiquitin chains in vitro. RIG-I-2CARD-mediated IFN-β reporter gene expression was abrogated by PLPs of all members of the Arterivirus family tested in the study, and further abolished RIG-I-2CARD-induced ubiquitination when PLP was overexpressed [59]. Nairoviruses of the family *Bunyaviridae* also possess DUB activity via their ovarian tumor (OTU)-like cysteine protease domain, which is located within the L protein along with the RNA-dependent RNA polymerase (RdRp), and the Crimean–Congo hemorrhagic fever virus (CCHFV) L protein also reduces RIG-I-mediated K63-linked ubiquitination [60] (Figure 1).

Many coronaviruses encode viral enzymes that also deubiquitinate RIG-I. Porcine epidemic diarrhea virus (PEDV) in the *Coronaviridae* family encodes papain-like protease 2 (PLP2), which has DUB activity, targeting both K48- and K63-ubiquitin linkage types. The usage of PLP2 catalytic mutants (C1729A, H1888A, and D1901A) provided evidence that PLP2, a proven IFN antagonist, possesses direct DUB activity. In studies where these mutants were overexpressed, PLP2 blocked the ubiquitination not only of RIG-I, but also of STING, a critical adaptor protein in the intracellular DNA sensing pathway [61]. Overexpressed PLP from human coronavirus (HCoV) NL63 and severe acute respiratory syndrome coronavirus (SARS-CoV) reduced ubiquitinated forms of many immune signaling molecules, including RIG-I, STING, TBK1, and IRF3, which blunted the antiviral immune response [62]. Middle East respiratory syndrome (MERS)-CoV PLPs have DUB and deISGylase activities when overexpressed, although their specific substrates and relative contributions of these activities during viral infection still remain unclear [63].

Studies have shown that certain members of the family *Picornaviridae*, which are positive-sense, single-stranded RNA viruses, can also deubiquitinate RIG-I, indicating that this mechanism is conserved amongst a large range of viruses. Seneca Valley virus (SVV) encodes the protease 3C protease (3C^pro^) that possesses DUB activity both when overexpressed and during native infection. 3C^pro^ acts to remove both K48- and K63-linked polyubiquitin chains from multiple substrates including RIG-I, TBK1, and TNF receptor associated factor 3 (TRAF3), thereby blocking downstream antiviral gene expression. The authors also identified that the amino acid residues 48 and 160 were integral to the DUB activity and viral replication [64]. Foot-and-mouth disease (FMDV) leader proteinase (L^pro^) is another PLP that inhibits both K48- and K63-linked ubiquitination in vitro and in overexpressed HEK293T cell-based assays. Additionally, the L^pro^ deubiquitination of RIG-I, TANK1, TRAF6, and TRAF3 negatively regulated type I IFN induction, although the exact ubiquitin-linkage types that were removed were not determined. Catalytic activity (at amino acids C51, D163/D164, and I83) of FMDV L^pro^ is required for efficient deubiquitination [65].

Besides HCV, other hepatitis viruses also evade RIG-I-mediated immune signaling, with differing mechanisms of action. Hepatitis B virus (HBV) X protein (HBx) deubiquitinates a number of substrates modified with K63-linked polyubiquitin including RIG-I and TRAF3, thereby negatively regulating type I IFN production [66]. Hepatitis E virus (HEV) encodes a papain-like cysteine protease (PCP), methyltransferase (MetT), RNA helicase, and RdRp that make up the open reading frame 1 (ORF1) polyprotein and inhibit poly(I:C)-induced IFN induction. It was previously reported that purified MetT-PCP protein has DUB activity in a cell-free assay [67]. Using an HEV replicon in hepatoma cells, the authors corroborated this study and demonstrated reduced K63-linked ubiquitination of RIG-I and TBK1 [68].

RIG-I has recently been discovered to detect DNA viruses such as adenoviruses, HSV-1, KSHV, and EBV, in addition to RNA viruses. During DNA virus infection, RIG-I was shown to recognize RNA polymerase III-generated viral transcripts or host-derived RNAs that are unshielded, mislocalized, or misprocessed [5,69,70]. Given the importance of RIG-I in the detection of DNA viruses, it is not surprising that these viruses have evolved effective ways to counteract RIG-I. KSHV encodes a viral DUB, ORF64, effective at removing both K48-linked and K63-linked ubiquitin chains both in vitro and in HEK293 cell-based assays [71]. ORF64 was discovered to specifically reduce RIG-I ubiquitination. The authors of this study utilized an enzymatically-defective ORF64 mutant (C29G) to demonstrate that the viral enzyme actively removes the K63-linked ubiquitination of the RIG-I 2CARD, and that TRIM25 overexpression restored RIG-I CARD ubiquitination. RIG-I depletion by short hairpin RNA (shRNA) resulted in increased KSHV *Rta* and *orf25* mRNA amounts, strengthening that RIG-I-mediated innate immune signaling plays a crucial role in suppressing KHSV lytic reactivation [72] (Figure 1).

### 2.3. Viral Control of Host DUB or E3 Ligase Expression

Yet another strategy that viruses have evolved to evade the RLR response is to manipulate the gene expression of critical host regulatory enzymes for RLR signaling, in particular DUBs or the E3 ligase TRIM25. Some viruses do so by manipulating the expression of specific microRNAs (miRNAs).

In the case of enterovirus 71 (EV71), its 3C protease (3C^pro^) downregulates miR-526, which positively regulates antiviral type I IFN responses. In a screen of 168 miRNAs that regulate IFN induction, miR-526 emerged as a top candidate. It was shown that miR-526 targets CYLD, a DUB that removes K63-linked ubiquitin chains from RIG-I to inactivate the immune response, for degradation [73]. EV71 3C^pro^ overexpression, or native infection of EV71, reduces miR-526 expression, which increases the abundance of CYLD and ultimately suppresses RIG-I activation [74].

Red spotted grouper nervous necrosis virus (RGNNV), a major pathogen of fish, induces expression of miR-202-5p, one of the top ten most upregulated miRNAs in this study. Antiviral gene expression (*IFN1*, *PKR*, *MXa*, and *ISG15*) was inhibited in RGNNV-infected cells while, conversely, viral replication was enhanced both in vitro and in zebrafish (zb). The authors found that the mechanism of viral evasion was that miR-202-5p targeted zbTRIM25 to inhibit zbTRIM25-mediated zbRIG-I CARD K63-ubiquitination [75], indicating that viral evasion of TRIM25-mediated RIG-I ubiquitination is also conserved in fish.

USP27X is a negative regulator of IFN-β expression induced by Sendai virus (SeV) infection, and its knockdown increases antiviral signaling following SeV or vesicular stomatitis mutant virus (VSV Δ51). The RIG-I CARDs interacted with USP27X during immunoprecipitation, which was enhanced during SeV infection. Additionally, ubiquitination assays demonstrated that USP27X reduced RIG-I ubiquitination, specifically the K63-ubiquitin linkage type. This was further substantiated through the use of *USP27X* knockout cells, in which RIG-I K63-linked ubiquitination levels induced by SeV infection were higher than those in control cells [76]. In the same study, it was shown that USP27X also regulates MDA5 by reducing K63-linked, but not K48-linked, ubiquitin chains on MDA5. Of note, ubiquitination of MDA5 is less well established than that of RIG-I, but multiple E3 ligases, TRIM40 and TRIM65, reportedly regulate MDA5 activity [26,27,77].

### 2.4. Viral Induction of Degradative K48-Linked Ubiquitination of RLRs and Signaling Molecules

Viruses also can escape detection by RLRs by inducing their degradation. Conceptually, there are two ways of how viruses achieve this: either through direct degradation of RLRs, or by enhancing the expression of cellular proteins that then degrade RLRs.

Toscana virus (TOSV) is a sandfly-borne virus whose NSs protein has E3 ubiquitin ligase activity, transferring ubiquitin from the charged E2 enzyme to the RIG-I CARDs and promoting proteasome-dependent proteolysis [78]. In vitro biochemical assay revealed that full length NSs is required for functional E3 ubiquitin ligase activity. TOSV NSs shares sequence homology to the NSs from sandfly fever Naples virus (SFNV) (which is also of the genus *Phlebovirus*), but SFNV NSs lacks the last 78 amino acids that are important for IFN antagonism and RIG-I degradation [79] (Figure 1).

The EBV-encoded tegument protein BPLF1 acts as a viral deconjugase and forms a complex with 14-3-3 and TRIM25 [80], promoting auto-ubiquitination and de-ubiquitination of TRIM25. During overexpression of BPLF1, or induction of AGS-Bx1 EBV-positive cells, TRIM25 becomes sequestered into protein aggregates, which also requires 14-3-3 and facilitates inhibition of the IFN response. BPLF1 activates TRIM25 by promoting a conformational rearrangement that mimics substrate binding, then attaches K48-linked ubiquitin chains and degrades TRIM25 [80].

Siglec-G is a lectin family member that recruits SHP2 and the E3 ubiquitin ligase c-Cbl to RIG-I. Upon viral infection or when overexpressed in HEK293T cells or RAW264.7 macrophages, Siglec-G is activated and recruits the phosphorylation of SHP2 and c-Cbl. RIG-I is also recruited and interacts with this complex and becomes degraded. c-Cbl conjugates K48-linked ubiquitination of RIG-I at the K813 residue for proteasomal degradation, while it does not affect the K63-ubiquitin linkage types. In the context of viral infection, Siglec-G inhibits VSV- and SeV-mediated IFN production, but not that mediated by HSV, and it does so by negatively regulating the RIG-I pathway [81].

## 3. De-ISGylation by SARS-CoV-2 PLpro

Although there are numerous studies that have demonstrated that viruses can evade type I IFN signaling via deISGylation, no mechanism has been identified related to RLR signaling until recently [63,82,83,84]. A new role for ISGylation has been discovered for the regulation of MDA5-mediated innate immunity [22]. The MDA5 CARDs are ISGylated at residues K23 and K43, which promotes MDA5 activation via the organization of MDA5 oligomers. Mutations at these sites abrogated MDA5 CARD ISGylation and MDA5-mediated cytokine induction. The conjugation of ISG15 to MDA5 upon virus infection serves as a trigger to promote MDA5 oligomerization and antiviral immunity, suggesting that MDA5 ISGylation serves an analogous role to the K63-linked ubiquitination of RIG-I. This study also showed that ISGylation is essential for the restriction of a range of viruses such as EMCV, DENV, and Zika virus. Although SARS-CoV-2, whose RNA is sensed by MDA5, was also restricted by MDA5 in an ISG15-dependent manner, the restricting activity against SARS-CoV-2 was much less than that seen for the other viruses tested. This result suggested that SARS-CoV-2 has evolved to antagonize the ISG15-dependent activation of MDA5. Indeed, SARS-CoV-2 papain-like protease (denoted as PLpro here), which is part of Nsp3, interacted with the MDA5 CARDs and antagonized MDA5 signaling via its deISGylating activity (Figure 1). An interaction of endogenous MDA5 and SARS-CoV-2 Nsp3 was also observed during native SARS-CoV-2 infection of A549-hACE2 cells. Suppression of MDA5 CARD ISGylation by PLpro was dependent on the catalytic site C111, and on one of the two ISG15-binding interfaces containing the residues R166 and E167. The mechanism defined here is MDA5-specific as SARS-CoV-2 PLpro did not interact with RIG-I, nor did it suppress RIG-I ubiquitination. This study proposes a model in which the SARS-CoV-2 Nsp3 protein interacts with the sensor MDA5 close to the viral RNA export site on the coronavirus replication organelle (i.e., double-membrane vesicles) and inhibits its CARD-dependent signaling function. Interestingly, the PLP enzymes of other coronaviruses such as SARS-CoV, MERS-CoV, murine hepatitis virus (MHV), and HCoV-NL63 were all able to bind to the MDA5 CARDs and to antagonize their ISGylation [22], suggesting that this immune evasion mechanism is widely conserved among coronaviruses, which warrants further investigation using authentic infection with these coronaviruses (Figure 1).

Some viral proteins also indirectly suppress MDA5 ISGylation, specifically through the manipulation of MDA5 CARD phosphorylation. Upon dsRNA recognition, MDA5 interacts with the phosphatase PP1, which subsequently dephosphorylates the MDA5 2CARD at S88 in a process essential for innate immune activation [85]. This in turn is antagonized by the V proteins of measles (MeV) and Nipah (NiV) viruses that bind to PP1α/γ to block MDA5 S88 dephosphorylation and thereby IFN induction [45]. Recently it was shown that V protein-mediated dysregulation of S88 phosphorylation was accompanied by reduced MDA5 ISGylation [22].

Downstream of RLRs (and also other sensors), SARS-CoV-2 PLpro cleaves ISG15 from IRF3 to inhibit antiviral type I IFN responses [86]. This, along with the finding on deISGylation of MDA5 by SARS-CoV-2 PLpro [22], suggests that ISGylation rather than ubiquitination could be the defining factor in effective SARS-CoV-2 restriction. Researchers are currently exploring the use of PLP inhibitors to block SARS-CoV-2 replication [87,88,89,90,91] and restore antiviral innate immune responses, which is an exciting avenue of research. Future studies will also determine the physiological relevance of PLP deISGylation for immune evasion and pathogenicity in suitable animal models [22,85].

Currently, SARS-CoV-2 PLpro is the only viral enzyme known to regulate ISGylation in the context of RLR-mediated innate immune signaling; however, as mentioned briefly, other coronavirus PLPs as well as OTU domain-containing viral proteins have been characterized to possess deISGylating activity (Reviewed in [58]). The PLPs from MERS-CoV, HCoV-NL63, EAV, and PRRSV have all been shown to have deISGylating activity in vitro. Coexpression of HCoV-NL63 PLP2 or MERS-CoV PLpro and ISG15 in HEK293T cells resulted in reduced ISGylation in a dose-dependent manner [63,92], while infection of monkey kidney cells with PRRSV was able to do the same [83,92]. Additionally, ectopic expression of OTUs of CCHFV and other nairoviruses as well as arteriviruses can specifically deconjugate ISGylated proteins in a protease-dependent manner although some were more effective than others [93,94]. It will be interesting to test if any of these viral enzymes also regulate MDA5 ISGylation or other ISG15-dependent signaling steps of the RLR pathway.

## 4. Conclusions

Our current understanding of the RLR pathway and regulatory PTMs has grown appreciably in the past decade, and a better understanding of these processes is critical for the development of drugs to avoid uncontrolled inflammatory responses. On the other hand, boosting RLR responses through the fine-tuning of their PTMs could be an effective measure against a wide range of viruses, especially new viruses that emerge and those for which no vaccines or antivirals exist. A panacea for all viral infections is unlikely, so understanding the precise molecular mechanisms that each single virus utilizes to subvert immune detection may allow us to target and inhibit viral IFN antagonists and restore host antiviral responses. The identification and specific targeting of viral DUBs or deISGylases, and the modulation of the abundance of cellular RLR-regulatory enzymes or miRNAs that control their expression, are all potential opportunities for translating major discoveries into antiviral drugs. Other conceivable applications are to generate attenuated viruses devoid of specific protein functions that inhibit ubiquitin- or ISG15-dependent antiviral responses for their use in vaccines, or to develop inhibitors against viral DUB enzymes.

Furthermore, the role of ISGylation in RLR signaling is a new area of research and other viral proteins may be found to alter this PTM to avoid immune detection. As with viral dysregulation of the host ubiquitinome, identification of viral mechanisms that target critical ISGylation events could lead to the development of much needed antiviral treatments. Exciting advances in this and further understanding of the mechanistic roles of ISGylation in antiviral immunity are exciting avenues for future research. These findings may help not only in the fight against SARS-CoV-2, but could also be applied to other viruses for which no effective treatment exists.

## Data Availability

No new data were created or analyzed. Data sharing is not applicable to this review.

## References

[1] Chiang C., Gack M.U. (2017). Post-translational Control of Intracellular Pathogen Sensing Pathways. Trends Immunol..

[2] Goubau D., Deddouche S., e Sousa C.R. (2013). Cytosolic sensing of viruses. Immunity.

[3] Liu G., Gack M.U. (2020). Distinct and Orchestrated Functions of RNA Sensors in Innate Immunity. Immunity.

[4] Rehwinkel J., Gack M.U. (2020). RIG-I-like receptors: Their regulation and roles in RNA sensing. Nat. Rev. Immunol..

[5] Chiang J.J., Sparrer K.M.J., van Gent M., Lassig C., Huang T., Osterrieder N., Hopfner K.P., Gack M.U. (2018). Viral unmasking of cellular 5S rRNA pseudogene transcripts induces RIG-I-mediated immunity. Nat. Immunol..

[6] Kato H., Takeuchi O., Sato S., Yoneyama M., Yamamoto M., Matsui K., Uematsu S., Jung A., Kawai T., Ishii K.J. (2006). Differential roles of MDA5 and RIG-I helicases in the recognition of RNA viruses. Nature.

[7] Loo Y.M., Fornek J., Crochet N., Bajwa G., Perwitasari O., Martinez-Sobrido L., Akira S., Gill M.A., Garcia-Sastre A., Katze M.G. (2008). Distinct RIG-I and MDA5 signaling by RNA viruses in innate immunity. J. Virol..

[8] Sharma S., tenOever B.R., Grandvaux N., Zhou G.P., Lin R., Hiscott J. (2003). Triggering the interferon antiviral response through an IKK-related pathway. Science.

[9] Liu J., Qian C., Cao X. (2016). Post-Translational Modification Control of Innate Immunity. Immunity.

[10] Li J., Chai Q.Y., Liu C.H. (2016). The ubiquitin system: A critical regulator of innate immunity and pathogen-host interactions. Cell Mol. Immunol..

[11] Giraldo M.I., Hage A., van Tol S., Rajsbaum R. (2020). TRIM Proteins in Host Defense and Viral Pathogenesis. Curr. Clin. Microbiol. Rep..

[12] Hage A., Rajsbaum R. (2019). To TRIM or not to TRIM: The balance of host-virus interactions mediated by the ubiquitin system. J. Gen. Virol..

[13] Perng Y.C., Lenschow D.J. (2018). ISG15 in antiviral immunity and beyond. Nat. Rev. Microbiol..

[14] Villarroya-Beltri C., Guerra S., Sanchez-Madrid F. (2017). ISGylation-a key to lock the cell gates for preventing the spread of threats. J. Cell Sci..

[15] Zhang H.L., Ye H.Q., Liu S.Q., Deng C.L., Li X.D., Shi P.Y., Zhang B. (2017). West Nile Virus NS1 Antagonizes Interferon Beta Production by Targeting RIG-I and MDA5. J. Virol..

[16] Malakhov M.P., Malakhova O.A., Kim K.I., Ritchie K.J., Zhang D.E. (2002). UBP43 (USP18) specifically removes ISG15 from conjugated proteins. J. Biol. Chem..

[17] Ritchie K.J., Hahn C.S., Kim K.I., Yan M., Rosario D., Li L., de la Torre J.C., Zhang D.E. (2004). Role of ISG15 protease UBP43 (USP18) in innate immunity to viral infection. Nat. Med..

[18] Ketscher L., Hannss R., Morales D.J., Basters A., Guerra S., Goldmann T., Hausmann A., Prinz M., Naumann R., Pekosz A. (2015). Selective inactivation of USP18 isopeptidase activity in vivo enhances ISG15 conjugation and viral resistance. Proc. Natl. Acad. Sci. USA.

[19] Eduardo-Correia B., Martinez-Romero C., Garcia-Sastre A., Guerra S. (2014). ISG15 is counteracted by vaccinia virus E3 protein and controls the proinflammatory response against viral infection. J. Virol..

[20] Werneke S.W., Schilte C., Rohatgi A., Monte K.J., Michault A., Arenzana-Seisdedos F., Vanlandingham D.L., Higgs S., Fontanet A., Albert M.L. (2011). ISG15 is critical in the control of Chikungunya virus infection independent of UbE1L mediated conjugation. PLoS Pathog.

[21] Zhang X., Bogunovic D., Payelle-Brogard B., Francois-Newton V., Speer S.D., Yuan C., Volpi S., Li Z., Sanal O., Mansouri D. (2015). Human intracellular ISG15 prevents interferon-alpha/beta over-amplification and auto-inflammation. Nature.

[22] Liu G., Lee J.H., Parker Z.M., Acharya D., Chiang J.J., van Gent M., Riedl W., Davis-Gardner M.E., Wies E., Chiang C. (2020). ISG15-dependent Activation of the RNA Sensor MDA5 and its Antagonism by the SARS-CoV-2 papain-like protease. bioRxiv.

[23] Choi S.J., Lee H.C., Kim J.H., Park S.Y., Kim T.H., Lee W.K., Jang D.J., Yoon J.E., Choi Y.I., Kim S. (2016). HDAC6 regulates cellular viral RNA sensing by deacetylation of RIG-I. EMBO J..

[24] He S., Zhao J., Song S., He X., Minassian A., Zhou Y., Zhang J., Brulois K., Wang Y., Cabo J. (2015). Viral pseudo-enzymes activate RIG-I via deamidation to evade cytokine production. Mol. Cell.

[25] Liu H.M., Jiang F., Loo Y.M., Hsu S., Hsiang T.Y., Marcotrigiano J., Gale M. (2016). Regulation of Retinoic Acid Inducible Gene-I (RIG-I) Activation by the Histone Deacetylase 6. EBioMedicine.

[26] Lang X., Tang T., Jin T., Ding C., Zhou R., Jiang W. (2017). TRIM65-catalized ubiquitination is essential for MDA5-mediated antiviral innate immunity. J. Exp. Med..

[27] Zhao C., Jia M., Song H., Yu Z., Wang W., Li Q., Zhang L., Zhao W., Cao X. (2017). The E3 Ubiquitin Ligase TRIM40 Attenuates Antiviral Immune Responses by Targeting MDA5 and RIG-I. Cell Rep..

[28] Takeuchi O., Akira S. (2009). Innate immunity to virus infection. Immunol. Rev..

[29] Chan Y.K., Gack M.U. (2016). Viral evasion of intracellular DNA and RNA sensing. Nat. Rev. Microbiol..

[30] Bowie A.G., Unterholzner L. (2008). Viral evasion and subversion of pattern-recognition receptor signalling. Nat. Rev. Immunol..

[31] Christensen M.H., Paludan S.R. (2017). Viral evasion of DNA-stimulated innate immune responses. Cell. Mol. Immunol..

[32] Eaglesham J.B., Kranzusch P.J. (2020). Conserved strategies for pathogen evasion of cGAS-STING immunity. Curr. Opin. Immunol..

[33] Gack M.U., Kirchhofer A., Shin Y.C., Inn K.S., Liang C., Cui S., Myong S., Ha T., Hopfner K.P., Jung J.U. (2008). Roles of RIG-I N-terminal tandem CARD and splice variant in TRIM25-mediated antiviral signal transduction. Proc. Natl. Acad. Sci. USA.

[34] Gack M.U., Shin Y.C., Joo C.H., Urano T., Liang C., Sun L., Takeuchi O., Akira S., Chen Z., Inoue S. (2007). TRIM25 RING-finger E3 ubiquitin ligase is essential for RIG-I-mediated antiviral activity. Nature.

[35] Sanchez J.G., Chiang J.J., Sparrer K.M.J., Alam S.L., Chi M., Roganowicz M.D., Sankaran B., Gack M.U., Pornillos O. (2016). Mechanism of TRIM25 Catalytic Activation in the Antiviral RIG-I Pathway. Cell Rep..

[36] Choudhury N.R., Heikel G., Trubitsyna M., Kubik P., Nowak J.S., Webb S., Granneman S., Spanos C., Rappsilber J., Castello A. (2017). RNA-binding activity of TRIM25 is mediated by its PRY/SPRY domain and is required for ubiquitination. BMC Biol..

[37] Haubrich K., Augsten S., Simon B., Masiewicz P., Perez K., Lethier M., Rittinger K., Gabel F., Cusack S., Hennig J. (2020). RNA binding regulates TRIM25-mediated RIG-I ubiquitylation. bioRxiv.

[38] Sanchez J.G., Sparrer K.M.J., Chiang C., Reis R.A., Chiang J.J., Zurenski M.A., Wan Y., Gack M.U., Pornillos O. (2018). TRIM25 Binds RNA to Modulate Cellular Anti-viral Defense. J. Mol. Biol..

[39] Gack M.U., Albrecht R.A., Urano T., Inn K.S., Huang I.C., Carnero E., Farzan M., Inoue S., Jung J.U., Garcia-Sastre A. (2009). Influenza A virus NS1 targets the ubiquitin ligase TRIM25 to evade recognition by the host viral RNA sensor RIG-I. Cell Host Microbe.

[40] Koliopoulos M.G., Lethier M., van der Veen A.G., Haubrich K., Hennig J., Kowalinski E., Stevens R.V., Martin S.R., Reis e Sousa C., Cusack S. (2018). Molecular mechanism of influenza A NS1-mediated TRIM25 recognition and inhibition. Nat. Commun..

[41] Rajsbaum R., Albrecht R.A., Wang M.K., Maharaj N.P., Versteeg G.A., Nistal-Villan E., Garcia-Sastre A., Gack M.U. (2012). Species-specific inhibition of RIG-I ubiquitination and IFN induction by the influenza A virus NS1 protein. PLoS Pathog.

[42] Oshiumi H., Matsumoto M., Hatakeyama S., Seya T. (2009). Riplet/RNF135, a RING finger protein, ubiquitinates RIG-I to promote interferon-beta induction during the early phase of viral infection. J. Biol. Chem..

[43] Ban J., Lee N.R., Lee N.J., Lee J.K., Quan F.S., Inn K.S. (2018). Human Respiratory Syncytial Virus NS 1 Targets TRIM25 to Suppress RIG-I Ubiquitination and Subsequent RIG-I-Mediated Antiviral Signaling. Viruses.

[44] Andrejeva J., Childs K.S., Young D.F., Carlos T.S., Stock N., Goodbourn S., Randall R.E. (2004). The V proteins of paramyxoviruses bind the IFN-inducible RNA helicase, mda-5, and inhibit its activation of the IFN-beta promoter. Proc. Natl. Acad. Sci. USA.

[45] Davis M.E., Wang M.K., Rennick L.J., Full F., Gableske S., Mesman A.W., Gringhuis S.I., Geijtenbeek T.B., Duprex W.P., Gack M.U. (2014). Antagonism of the phosphatase PP1 by the measles virus V protein is required for innate immune escape of MDA5. Cell Host Microbe.

[46] Rodriguez K.R., Horvath C.M. (2013). Amino acid requirements for MDA5 and LGP2 recognition by paramyxovirus V proteins: A single arginine distinguishes MDA5 from RIG-I. J. Virol..

[47] Sanchez-Aparicio M.T., Feinman L.J., Garcia-Sastre A., Shaw M.L. (2018). Paramyxovirus V Proteins Interact with the RIG-I/TRIM25 Regulatory Complex and Inhibit RIG-I Signaling. J. Virol..

[48] Min Y.Q., Ning Y.J., Wang H., Deng F. (2020). A RIG-I-like receptor directs antiviral responses to a bunyavirus and is antagonized by virus-induced blockade of TRIM25-mediated ubiquitination. J. Biol. Chem..

[49] Oshiumi H., Miyashita M., Matsumoto M., Seya T. (2013). A distinct role of Riplet-mediated K63-Linked polyubiquitination of the RIG-I repressor domain in human antiviral innate immune responses. PLoS Pathog.

[50] Li X.D., Sun L., Seth R.B., Pineda G., Chen Z.J. (2005). Hepatitis C virus protease NS3/4A cleaves mitochondrial antiviral signaling protein off the mitochondria to evade innate immunity. Proc. Natl. Acad. Sci. USA.

[51] Meylan E., Curran J., Hofmann K., Moradpour D., Binder M., Bartenschlager R., Tschopp J. (2005). Cardif is an adaptor protein in the RIG-I antiviral pathway and is targeted by hepatitis C virus. Nature.

[52] Pijlman G.P., Funk A., Kondratieva N., Leung J., Torres S., van der Aa L., Liu W.J., Palmenberg A.C., Shi P.Y., Hall R.A. (2008). A highly structured, nuclease-resistant, noncoding RNA produced by flaviviruses is required for pathogenicity. Cell Host Microbe.

[53] Funk A., Truong K., Nagasaki T., Torres S., Floden N., Balmori Melian E., Edmonds J., Dong H., Shi P.Y., Khromykh A.A. (2010). RNA structures required for production of subgenomic flavivirus RNA. J. Virol..

[54] Slonchak A., Khromykh A.A. (2018). Subgenomic flaviviral RNAs: What do we know after the first decade of research. Antiviral Res..

[55] Pauli E.K., Chan Y.K., Davis M.E., Gableske S., Wang M.K., Feister K.F., Gack M.U. (2014). The ubiquitin-specific protease USP15 promotes RIG-I-mediated antiviral signaling by deubiquitylating TRIM25. Sci. Signal.

[56] Manokaran G., Finol E., Wang C., Gunaratne J., Bahl J., Ong E.Z., Tan H.C., Sessions O.M., Ward A.M., Gubler D.J. (2015). Dengue subgenomic RNA binds TRIM25 to inhibit interferon expression for epidemiological fitness. Science.

[57] Chiang C., Pauli E.K., Biryukov J., Feister K.F., Meng M., White E.A., Munger K., Howley P.M., Meyers C., Gack M.U. (2018). The Human Papillomavirus E6 Oncoprotein Targets USP15 and TRIM25 To Suppress RIG-I-Mediated Innate Immune Signaling. J. Virol..

[58] Mielech A.M., Chen Y., Mesecar A.D., Baker S.C. (2014). Nidovirus papain-like proteases: Multifunctional enzymes with protease, deubiquitinating and deISGylating activities. Virus Res..

[59] van Kasteren P.B., Beugeling C., Ninaber D.K., Frias-Staheli N., van Boheemen S., Garcia-Sastre A., Snijder E.J., Kikkert M. (2012). Arterivirus and nairovirus ovarian tumor domain-containing Deubiquitinases target activated RIG-I to control innate immune signaling. J. Virol..

[60] Scholte F.E.M., Zivcec M., Dzimianski J.V., Deaton M.K., Spengler J.R., Welch S.R., Nichol S.T., Pegan S.D., Spiropoulou C.F., Bergeron E. (2017). Crimean-Congo Hemorrhagic Fever Virus Suppresses Innate Immune Responses via a Ubiquitin and ISG15 Specific Protease. Cell Rep..

[61] Xing Y., Chen J., Tu J., Zhang B., Chen X., Shi H., Baker S.C., Feng L., Chen Z. (2013). The papain-like protease of porcine epidemic diarrhea virus negatively regulates type I interferon pathway by acting as a viral deubiquitinase. J. Gen. Virol..

[62] Sun L., Xing Y., Chen X., Zheng Y., Yang Y., Nichols D.B., Clementz M.A., Banach B.S., Li K., Baker S.C. (2012). Coronavirus papain-like proteases negatively regulate antiviral innate immune response through disruption of STING-mediated signaling. PLoS ONE.

[63] Mielech A.M., Kilianski A., Baez-Santos Y.M., Mesecar A.D., Baker S.C. (2014). MERS-CoV papain-like protease has deISGylating and deubiquitinating activities. Virology.

[64] Xue Q., Liu H., Zhu Z., Yang F., Xue Q., Cai X., Liu X., Zheng H. (2018). Seneca Valley Virus 3C protease negatively regulates the type I interferon pathway by acting as a viral deubiquitinase. Antivir. Res..

[65] Wang D., Fang L., Li P., Sun L., Fan J., Zhang Q., Luo R., Liu X., Li K., Chen H. (2011). The leader proteinase of foot-and-mouth disease virus negatively regulates the type I interferon pathway by acting as a viral deubiquitinase. J. Virol..

[66] Jiang J., Tang H. (2010). Mechanism of inhibiting type I interferon induction by hepatitis B virus X protein. Protein Cell.

[67] Karpe Y.A., Lole K.S. (2011). Deubiquitination activity associated with hepatitis E virus putative papain-like cysteine protease. J. Gen. Virol..

[68] Nan Y., Yu Y., Ma Z., Khattar S.K., Fredericksen B., Zhang Y.J. (2014). Hepatitis E virus inhibits type I interferon induction by ORF1 products. J. Virol..

[69] Ablasser A., Bauernfeind F., Hartmann G., Latz E., Fitzgerald K.A., Hornung V. (2009). RIG-I-dependent sensing of poly(dA:dT) through the induction of an RNA polymerase III-transcribed RNA intermediate. Nat. Immunol..

[70] Zhang Y., Dittmer D.P., Mieczkowski P.A., Host K.M., Fusco W.G., Duncan J.A., Damania B. (2018). RIG-I Detects Kaposi’s Sarcoma-Associated Herpesvirus Transcripts in a RNA Polymerase III-Independent Manner. mBio.

[71] Gonzalez C.M., Wang L., Damania B. (2009). Kaposi’s sarcoma-associated herpesvirus encodes a viral deubiquitinase. J. Virol..

[72] Inn K.S., Lee S.H., Rathbun J.Y., Wong L.Y., Toth Z., Machida K., Ou J.H., Jung J.U. (2011). Inhibition of RIG-I-mediated signaling by Kaposi’s sarcoma-associated herpesvirus-encoded deubiquitinase ORF64. J. Virol..

[73] Friedman C.S., O’Donnell M.A., Legarda-Addison D., Ng A., Cardenas W.B., Yount J.S., Moran T.M., Basler C.F., Komuro A., Horvath C.M. (2008). The tumour suppressor CYLD is a negative regulator of RIG-I-mediated antiviral response. EMBO Rep..

[74] Xu C., He X., Zheng Z., Zhang Z., Wei C., Guan K., Hou L., Zhang B., Zhu L., Cao Y. (2014). Downregulation of microRNA miR-526a by enterovirus inhibits RIG-I-dependent innate immune response. J. Virol..

[75] Liu W., Jin Y., Zhang W., Xiang Y., Jia P., Yi M., Jia K. (2020). MiR-202-5p Inhibits RIG-I-Dependent Innate Immune Responses to RGNNV Infection by Targeting TRIM25 to Mediate RIG-I Ubiquitination. Viruses.

[76] Tao X., Chu B., Xin D., Li L., Sun Q. (2020). USP27X negatively regulates antiviral signaling by deubiquitinating RIG-I. PLoS Pathog.

[77] Jiang X., Kinch L.N., Brautigam C.A., Chen X., Du F., Grishin N.V., Chen Z.J. (2012). Ubiquitin-induced oligomerization of the RNA sensors RIG-I and MDA5 activates antiviral innate immune response. Immunity.

[78] Gori Savellini G., Anichini G., Gandolfo C., Prathyumnan S., Cusi M.G. (2019). Toscana virus non-structural protein NSs acts as E3 ubiquitin ligase promoting RIG-I degradation. PLoS Pathog.

[79] Gori-Savellini G., Valentini M., Cusi M.G. (2013). Toscana virus NSs protein inhibits the induction of type I interferon by interacting with RIG-I. J. Virol..

[80] Gupta S., Yla-Anttila P., Sandalova T., Sun R., Achour A., Masucci M.G. (2019). 14-3-3 scaffold proteins mediate the inactivation of trim25 and inhibition of the type I interferon response by herpesvirus deconjugases. PLoS Pathog.

[81] Chen W., Han C., Xie B., Hu X., Yu Q., Shi L., Wang Q., Li D., Wang J., Zheng P. (2013). Induction of Siglec-G by RNA viruses inhibits the innate immune response by promoting RIG-I degradation. Cell.

[82] Medina G.N., Azzinaro P., Ramirez-Medina E., Gutkoska J., Fang Y., Diaz-San Segundo F., de Los Santos T. (2020). Impairment of the DeISGylation Activity of Foot-and-Mouth Disease Virus Lpro Causes Attenuation In Vitro and In Vivo. J. Virol..

[83] Sun Z., Li Y., Ransburgh R., Snijder E.J., Fang Y. (2012). Nonstructural protein 2 of porcine reproductive and respiratory syndrome virus inhibits the antiviral function of interferon-stimulated gene 15. J. Virol..

[84] Zimmermann C., Buscher N., Krauter S., Kramer N., Wolfrum U., Sehn E., Tenzer S., Plachter B. (2018). The Abundant Tegument Protein pUL25 of Human Cytomegalovirus Prevents Proteasomal Degradation of pUL26 and Supports Its Suppression of ISGylation. J. Virol..

[85] Wies E., Wang M.K., Maharaj N.P., Chen K., Zhou S., Finberg R.W., Gack M.U. (2013). Dephosphorylation of the RNA sensors RIG-I and MDA5 by the phosphatase PP1 is essential for innate immune signaling. Immunity.

[86] Shin D., Mukherjee R., Grewe D., Bojkova D., Baek K., Bhattacharya A., Schulz L., Widera M., Mehdipour A.R., Tascher G. (2020). Papain-like protease regulates SARS-CoV-2 viral spread and innate immunity. Nature.

[87] Freitas B.T., Durie I.A., Murray J., Longo J.E., Miller H.C., Crich D., Hogan R.J., Tripp R.A., Pegan S.D. (2020). Characterization and Noncovalent Inhibition of the Deubiquitinase and deISGylase Activity of SARS-CoV-2 Papain-Like Protease. ACS Infect. Dis..

[88] Klemm T., Ebert G., Calleja D.J., Allison C.C., Richardson L.W., Bernardini J.P., Lu B.G., Kuchel N.W., Grohmann C., Shibata Y. (2020). Mechanism and inhibition of the papain-like protease, PLpro, of SARS-CoV-2. EMBO J..

[89] Pitsillou E., Liang J., Karagiannis C., Ververis K., Darmawan K.K., Ng K., Hung A., Karagiannis T.C. (2020). Interaction of small molecules with the SARS-CoV-2 main protease in silico and in vitro validation of potential lead compounds using an enzyme-linked immunosorbent assay. Comput. Biol. Chem..

[90] Ratia K., Pegan S., Takayama J., Sleeman K., Coughlin M., Baliji S., Chaudhuri R., Fu W., Prabhakar B.S., Johnson M.E. (2008). A noncovalent class of papain-like protease/deubiquitinase inhibitors blocks SARS virus replication. Proc. Natl. Acad. Sci. USA.

[91] Rut W., Lv Z., Zmudzinski M., Patchett S., Nayak D., Snipas S.J., El Oualid F., Huang T.T., Bekes M., Drag M. (2020). Activity profiling and crystal structures of inhibitor-bound SARS-CoV-2 papain-like protease: A framework for anti-COVID-19 drug design. Sci. Adv..

[92] Clementz M.A., Chen Z., Banach B.S., Wang Y., Sun L., Ratia K., Baez-Santos Y.M., Wang J., Takayama J., Ghosh A.K. (2010). Deubiquitinating and interferon antagonism activities of coronavirus papain-like proteases. J. Virol..

[93] Akutsu M., Ye Y., Virdee S., Chin J.W., Komander D. (2011). Molecular basis for ubiquitin and ISG15 cross-reactivity in viral ovarian tumor domains. Proc. Natl. Acad. Sci. USA.

[94] Frias-Staheli N., Giannakopoulos N.V., Kikkert M., Taylor S.L., Bridgen A., Paragas J., Richt J.A., Rowland R.R., Schmaljohn C.S., Lenschow D.J. (2007). Ovarian tumor domain-containing viral proteases evade ubiquitin- and ISG15-dependent innate immune responses. Cell Host Microbe.

